# Validation of Induced Microglia-Like Cells (iMG Cells) for Future Studies of Brain Diseases

**DOI:** 10.3389/fncel.2021.629279

**Published:** 2021-04-09

**Authors:** Atoshi Banerjee, Yimei Lu, Kenny Do, Travis Mize, Xiaogang Wu, Xiangning Chen, Jingchun Chen

**Affiliations:** ^1^Nevada Institute of Personalized Medicine, University of Nevada, Las Vegas, NV, United States; ^2^Department of Psychology, Nevada Institute of Personalized Medicine, University of Nevada, Las Vegas, NV, United States; ^3^Department of Genomic Medicine, The University of Texas MD Anderson Cancer Center, Houston, TX, United States; ^4^410 AI, LLC, Germantown, MD, United States

**Keywords:** induced microglia-like cells (iMG cells), RNA-seq profile, peripheral blood mononuclear cells, phagocytosis, schizophrenia, Alzheimer's disease, synaptic pruning

## Abstract

Microglia are the primary resident immune cells of the central nervous system that maintain physiological homeostasis in the brain and contribute to the pathogenesis of many psychiatric disorders and neurodegenerative diseases. Due to the lack of appropriate human cellular models, it is difficult to study the basic pathophysiological processes linking microglia to brain diseases. In this study, we adopted a microglia-like cellular model derived from peripheral blood monocytes with granulocyte-macrophage colony-stimulating factor (GM-CSF) and interleukin-34 (IL-34). We characterized and validated this *in vitro* cellular model by morphology, immunocytochemistry, gene expression profiles, and functional study. Our results indicated that the iMG cells developed typical microglial ramified morphology, expressed microglial specific surface markers (P2RY12 and TMEM119), and possessed phagocytic activity. Principal component analyses and multidimensional scaling analyses of RNA-seq data showed that iMG cells were distinct from monocytes and induced macrophages (iMacs) but clustered closer to human microglia and hiPSC-induced microglia. Heatmap analyses also found that iMG cells, but not monocytes, were closely clustered with human primary microglia. Further pathway and relative expression analysis indicated that unique genes from iMG cells were involved in the regulation of the complement system, especially in the synapse and ion transport. Overall, our data demonstrated that the iMG model mimicked many features of the brain resident microglia, highlighting its utility in the study of microglial function in many brain diseases, such as schizophrenia and Alzheimer's disease (AD).

## Introduction

Microglia are the primary immune cells of the central nervous system (CNS) that belong to the myeloid lineage. The origins of microglia and their functions in the CNS have been extensively debated since they were first described by Pio del Rio-Hortega in 1932 (Río-Hortega, 1932). In the mouse brain, microglia originate from myeloid progenitors in the yolk sac that migrate to the brain during early embryonic stages before the blood-brain barrier is formed (Ginhoux et al., 2013). A similar pattern of events may occur in humans, but under certain inflammatory conditions, the recruitment of bone marrow-derived progenitors may supplement the microglial population to some extent (Ginhoux and Prinz, 2015). The origins of human microglia are more heterogeneous, with one population from myeloid/mesenchymal origin in the yolk sac and a second population representing a developmental and transitory form of fetal macrophage (possibly monocytes). During normal brain development, microglia proliferate and self-renew continually throughout life to maintain cell numbers, without any contribution from bone marrow-derived macrophages (Elmore et al., 2014). Microglia serve a variety of important functions, including immune surveillance, neurogenesis, synaptic pruning, and phagocytosis (Stansley et al., 2012). Microglia-mediated synaptic loss by phagocytosis strongly correlates with cognitive decline in patients with schizophrenia or AD.

In response to environmental stimuli/stresses, microglia are highly plastic and can be activated into two distinctive phenotypes, the classical pro-inflammatory state (M1) and the alternative anti-inflammatory state (M2) (Ma et al., 2017). Microglial activation and neuroinflammation are hallmarks of many CNS pathologies. In response to lipopolysaccharide (LPS) and interferon (IFN)-γ, microglia are activated to pro-inflammatory M1 state and release cytokines, such as Interleukin (IL)-1β, IL-6, Tumor Necrosis Factor (TNF)-α, and neurotoxic factors. Prolonged hyperactivity of microglia may lead to neuronal apoptosis and brain damage, as seen in schizophrenia (Pérez-Neri et al., 2006; Monji et al., 2013) and AD (Akiyama et al., 2000; Heneka et al., 2015; Wang et al., 2015). Alternatively, in response to IL-4, microglia can be activated to the M2 state and release numerous anti-inflammatory factors, including CD206 and IL-10. M2 microglia promote healing and improve phagocytic capabilities (Cherry et al., 2014).

It is noteworthy that many studies have utilized a mouse model to investigate microglial phenotypes and functions. However, microglial transcriptomic signatures vary between mice and humans (Deczkowska et al., 2018; Friedman et al., 2018; Zhou et al., 2020); most animal-based clinical trials have failed in human diseases, including AD (Franco and Cedazo-Minguez, 2014). Other common approaches to study microglia in human diseases include post-mortem examination of the brain or brain imaging from living patients. Nevertheless, *in vitro* studies on human primary microglia are challenging due to technical and ethical issues. Human microglial cell lines, such as HMO6 cells (Nagai et al., 2005) and HMC3 cells (Janabi et al., 1995), can be an alternative model for human microglia. However, these cell lines have limited capacity in capturing disease- or patient-specific features of microglia. Furthermore, cell lines may change their cellular phenotypes and functions over time due to genetic manipulations and multiple passages.

Recently, many researchers are experimenting with different reprogramming methods to generate human induced pluripotent stem cell (hiPSC)-derived microglia (Muffat et al., 2016; Abud et al., 2017; Douvaras et al., 2017; Haenseler et al., 2017; Pandya et al., 2017; Takata et al., 2017). While research on hiPSC-derived microglia has been shown to be promising, the field still faces technical challenges, such as long differentiation period, which may not be suitable for high throughput clinical trials, especially for AD patients. Therefore, it is urgent to establish an efficient cellular model representing patient-specific microglia for neurological disease studies.

In this study, we generated and modified a human microglial model, with a 10–14-day conversion from human peripheral blood monocytes, according to previous studies (Ohgidani et al., 2014). The main purpose of this study was to validate the iMG cellular model with morphology, surface markers, functional assays, and RNA-seq profile. Our goals are (1) establish and validate a personalized microglia-like model from human monocytes; (2) develop an *in vitro* cellular system to study microglial functions, especially phagocytosis and polarization, at molecular and gene expression levels; (3) demonstrate a potential application in psychiatric and neurological diseases, such as schizophrenia and AD. Our data showed that iMG cells mimicked human primary microglia with their typical morphology, surface markers, phagocytic capacity, and plasticity for polarization. With whole-genome transcript profile, we also demonstrated that iMG cells were clustered closely with human microglia.

## Materials and Methods

### Materials

PBMCs were purchased from AllCells, LLC (Alameda, CA, USA, #PB003F and #PB001). Fresh blood was purchased from Gulf Coast Regional Blood Center. All other reagents were RPMI-1640 without glutamine (vWR, Radnor, PA, USA, #vWRL0106-0500), RPMI-1640 Glutamax (Life Technologies, Carlsbad, CA), Heat-inactivated fetal bovine serum (FBS, Endotoxin <1.0 EU/mL, Rockland Immunochemicals Inc, Pottstown, PA, #FBS-01-0100), Penicillin-streptomycin (P/S) (Gibco), cell culture plates (Corning, NY), Geltrex (Thermo Fisher Scientific Waltham, MA, #A1413202), Trizol (Invitrogen, Carlsbad, CA, #15596018), SuperScript™ IV VILO™ Master Mix (Invitrogen, Carlsbad, CA), High-Capacity cDNA Reverse Transcription Kit with RNase Inhibitor (Thermo Fisher Scientific Waltham, MA, #4374966), 2100 Bioanalyzer (Agilent Technologies, Santa Clara, CA), Phosphate buffer saline (PBS) (Genesee Scientific, San Diego, CA), Histopaque-1077 (Sigma Chemical Co., St. Louis, MO), SepMate™-50 tube (Stem cell technologies), IL-34 (R&D Systems Minneapolis, MN, #5265-IL-010/CF), GM-CSF (R&D Systems Minneapolis, MN, #215-GM-010/CF), Lipopolysaccharide (LPS) (Sigma, St. Louis, MO, #L6529), human IL-4 (Biolegend, San Diego, CA, #714904), DMSO (Sigma, St. Louis, MO), Paraformaldehyde (PFA) (Sigma, St. Louis, MO), Fluoromount-G™ Mounting Medium with DAPI (Invitrogen, Carlsbad, CA), human HiLyte™ Fluor 488-labeled Aβ protein (1–42) (Anaspec, #AS-60479-01), and hexafluoroisopropanol (HFIP) (#H8508, Sigma).

### Antibodies

Primary antibodies were anti-TMEM119 (Proteintech, #27585-1-AP), anti-P2RY12 (Alomone Labs, #APR-020), anti-Iba1 (Abcam, #ab15690), anti-PU.1 (Abcam, #ab88082), anti-CX3CR1 (Invitrogen, #PA5-19910), and anti-TREM2 (Abcam, #ab209814). Conjugated secondary antibodies were Alexa Fluor 488 (#A11034) and Alexa Fluor 594 (#A10036) from Invitrogen Carlsbad, CA.

### Generation of Induced Microglia-Like Cells (iMG cells) From Monocytes

Human peripheral blood mononuclear cells (PBMCs) were isolated from fresh blood by density gradient centrifugation with Histopaque-1077 in SepMate™-50 tubes. iMG cells were generated following the previously reported instructions with some modifications (Ohgidani et al., 2014). Briefly, 1 × 10^6^ cells/ml fresh PBMCs were first resuspended in complete media (RPMI-1640 containing 10% FBS and 1% P/S) in plates pre-coated with 2% Geltrex and incubated overnight at 37°C with 5% CO_2_. For frozen PBMCs, cells were thawed and washed with complete media before plating. The next day (Day 0), non-adherent cells were carefully removed. The adherent monocytes were cultured with serum-free media (RPMI-1640 Glutamax supplemented with 0.1 μg/ml IL-34, 0.01 μg/ml GM-CSF, and 1% P/S) for 10–14 days. For comparison, induced macrophages (iMacs) were also generated from monocytes by induction with RPMI-1640 Glutamax media containing 10% FBS, 0.01 μg/ml GM-CSF, and 1% P/S for 7 days. During the induction, we replaced fresh media every 3–4 days.

### Cell Morphology

During the induction, cellular morphology was monitored with phase-contrast microscopy (Accu-Scope EXI-300, ACCU-SCOPE Inc., Commack, NY, USA). Images were captured with an AU-300-HD digital camera (ACCU-SCOPE Inc.) through CaptaVision PC Imaging Software.

### Immunocytochemistry

For immunocytochemistry, iMG cells or iMacs were first fixed with 4% PFA for 10 min and washed thrice with PBS. For intracellular staining, cells were permeabilized with 0.01% tween 20/PBS for 30 min. Cells were then blocked with 5% BSA/PBS for 30 min, followed by incubation with primary antibody (RT for 1 h or 4°C overnight) and then secondary antibody (RT for 1 h at dark). The primary antibodies used in this study were anti-P2RY12 (1:200), anti-TREM2 (1:500), anti-CX3CR1 (1:1000), anti-Iba1 (1:300), anti-PU.1 (1:200), and anti-TMEM119 (1:500) diluted in blocking solution. Depending on the species of primary antibodies, 1:500 Alexa Fluor 488- or Fluor 594-conjugated antibodies were used for staining. Cells were then mounted on a glass slide with Fluoromount G containing DAPI. Images were taken with a confocal laser scanning microscope (Nikon A1R) equipped with the NIS-Elements Advanced Research software. Exposure settings within each set of experiments were kept constant for comparison.

### Fibrillary Amyloid-Beta (fAβ_42_) Preparation and Phagocytosis Assay

The preparation of fibrillary fluorescent Aβ was modified from the previously described procedure (Stine et al., 2003; Jungbauer et al., 2009). Briefly, Human HiLyte™ Fluor 488-labeled Aβ (1–42) was first resuspended as 0.1 mM HFIP and left to dry in a fume hood overnight. The resulting peptide film was stored (desiccated) at −20°C for later use. On the day before the experiment, the films were dissolved in 0.1 mM anhydrous DMSO and sonicated at 22°C for 10 min in a bath sonicator (Bioruptor Pico sonication device). To form fibrillary amyloid-beta 42 (fAβ_42_), the solution was diluted to 100 μM in 10 mM HCl with vigorous shaking at 37°C for 24 h. iMG cells were incubated with 100 nM fAβ_42_ in serum-free DMEM/F12 media at 37°C for 4 h. Cells were then quenched with 50 mM NH_4_Cl for 5 min and fixed with 4% PFA for 5 min at RT. Next, cells were stained with anti-Iba1 and mounted on a glass slide with Fluoromount G containing DAPI. Cells without fAβ_42_ treatment were used as negative controls for background setting. Fluorescent images were captured as described in the previous section.

Phagocytosis assay was also quantitated by flow cytometry using Sony SH800 (Sony Biotechnology). To this end, cells with or without fAβ_42_ incubation were first detached with 10 mM EDTA for 5 min and harvested by cell lifter. Cells were then fixed with 4% PFA and acquired by a flow cytometer using 488 laser and analyzed by Flowjo software (Tree Star, San Carlos, CA).

### Quantitative Real-Time Polymerase Chain Reaction (qRT-PCR) for *in vitro* Polarization

In this study, we used LPS and IL-4 for *in vitro* microglial activation and polarization. On Day 11, LPS (10 μg/ml) or IL-4 (20 ng/ml) were added to the iMG cells and incubated for 24 h at 37°C with 5% CO_2_. The next day, cell morphology was monitored and captured for each group. The total RNA was isolated using Trizol according to the manufacturer's instruction and subjected to cDNA synthesis using SuperScript™ IV VILO™ Master Mix. With the following primers, for M1 (*TNF-*α) and M2 (*CD206*) gene-specific primers, qRT-PCR was performed using PowerUp SYBR Green PCRmix. The relative expression was normalized to the housekeeping control gene β*-actin*. Fold changes in mRNA levels between stimulated and non-stimulated cells were calculated using the 2^−(Δ*ΔCT*)^ method.

The sequences of primers were as follows:

β*-actin F 5*′*-GATGCAGAAGGAGATCACTGC-3*′

β*-actin R 5*′*-ATACTCCTGCTTGCTGATCCA-3*′

*TNF-*α *F 5*′*-CCCAGGCAGTCAGATCATCTTCT-3*′

*TNF-*α *R 5*′*-ATGAGGTACAGGCCCTCTGAT-3*′

*CD206 F 5*′*-GTCATTCCGGGTGCTGTTCTCC-3*′

*CD206 R 5*′*-TTCGGCATCCTGGTTGCAAG-3*′

### RNA-seq Data Processing

For RNA-seq, total RNA was extracted from five subjects, including three monocytes, three iMacs, and four iMG cells, by RNeasy Mini kit (Qiagen) following the manufacturer's instruction. The subjects' information, including age, sex, etc., were described in Supplementary Table 1. RNA quality was assessed by Agilent 2100 Bioanalyzer. Total RNA with RIN > 8, 200 ng was subjected to cDNA library construction with RNA Library Prep Kit v2 protocol (Illumina, San Diego, CA). RNA-seq was performed on the polyadenylated fraction of RNA using Illumina NextSeq 500 platform at a depth of more than 20 million reads. FASTQ files from RNA-seq experiments were aligned to reference 1000 Genomes GRCh37 with STAR v2.5.2a (Dobin et al., 2013), and quality-controlled bam files were generated by GATK (v3.7 and v4.beta.2) RNA-seq pipeline (Al-Ars et al., 2020). Read counts from each transcript were finally collected using FeatureCounts v1.5.3 (Liao et al., 2014). The whole genome RNA-seq project PRJNA678841 generated during the current study in the format of fastq files have been deposited at the National Center for Biotechnology Information (NCBI) repository (http://www.ncbi.nlm.nih.gov/bioproject/678841).

### Principal Component Analysis (PCA), Multidimensional Scaling (MDS), and Hierarchical Clustering Analysis

To determine the uniformity among the cell types, we conducted PCA using the R package pcaExplorer to perform a simple overview of the counts from their expression profiles (Marini and Binder, 2019). To further determine how the iMG cells resemble the human microglia and hiPSC-induced microglia, we performed MDS analysis with microglia expression datasets from Muffat et al. (2016), Gosselin et al. (2017), and Grubman et al. (2020). Read counts were transformed to fragments per kilobase of transcript per million mapped reads (FPKM) with the edgeR v.3.22.3 (Robinson et al., 2010). Data were then normalized with the removebatcheffect function and rescaled with R scales package. For MDS calculation, we used cmdscale function in R stats and magrittr package (Stefan Milton et al., 2020). The MDS plot was generated with the R ggplot package (Warnes et al., 2020).

For hierarchical clustering analysis, we started with a total of 914 non-overlapped microglial signature genes, including the 881 genes from Gosselin et al.'s study (Gosselin et al., 2017) and 42 microglial genes from McQuade and Blurton-Jones's study (McQuade and Blurton-Jones, 2019) (See Supplementary Table 2). Variance stabilized transformation (VST) data obtained from DESeq2 v1.28.1 were used to process the two datasets (Love et al., 2014). From the 773 overlapped genes between our data and reference data, we compared the expression consistency with the formula for consistency scores = 2xy/(x^2^ + y^2^)^1/2^, where x and y represented the fold change (FC) from monocytes to microglia in the two datasets, respectively. Genes with consistency scores >0.50 were considered as consistent genes (312 genes). We then conducted differential expression analysis DESeq2, as mentioned above. Raw count data were normalized by the library size and log-transformation. Differentially expressed genes were estimated using negative binomial general model statistics. Heatmap analysis was conducted with the consistent genes that overlapped with the differentially expressed genes (201 genes). We used Heatmap.2 function in the R ggplot package (Warnes et al., 2020) for the Heatmap graph.

For Venn analysis, genes with a mean of the normalized counts >10 were assigned to each cell type. The Venn diagram for monocytes, iMacs, and iMG cells was generated according to the website (http://bioinformatics.psb.ugent.be/webtools/Venn/). For the unique genes obtained from the Venn diagram in each specific cell type, we further perform the gene enrichment analysis according to the instruction from Zhou et al.'s paper (Zhou et al., 2019).

Of particular interest, we further explored the gene regulation of some microglia-specific genes reported in Gosselin et al.'s study (Gosselin et al., 2017) or Ryan et al.'s study (Ryan et al., 2017). For this purpose, we used R package pcaExplorer to compare specific gene expression between iMG cells and monocytes from our sample and the sample from (Gosselin et al., 2017).

### Statistical Analyses

Statistical analyses for RT-PCR were conducted with Student's *t*-test or by ANNOVA for comparing more than two groups, and a *p*-value ≤ 0.05 were considered significant.

## Results

### Morphology of iMG Cells

**Figure 1:**
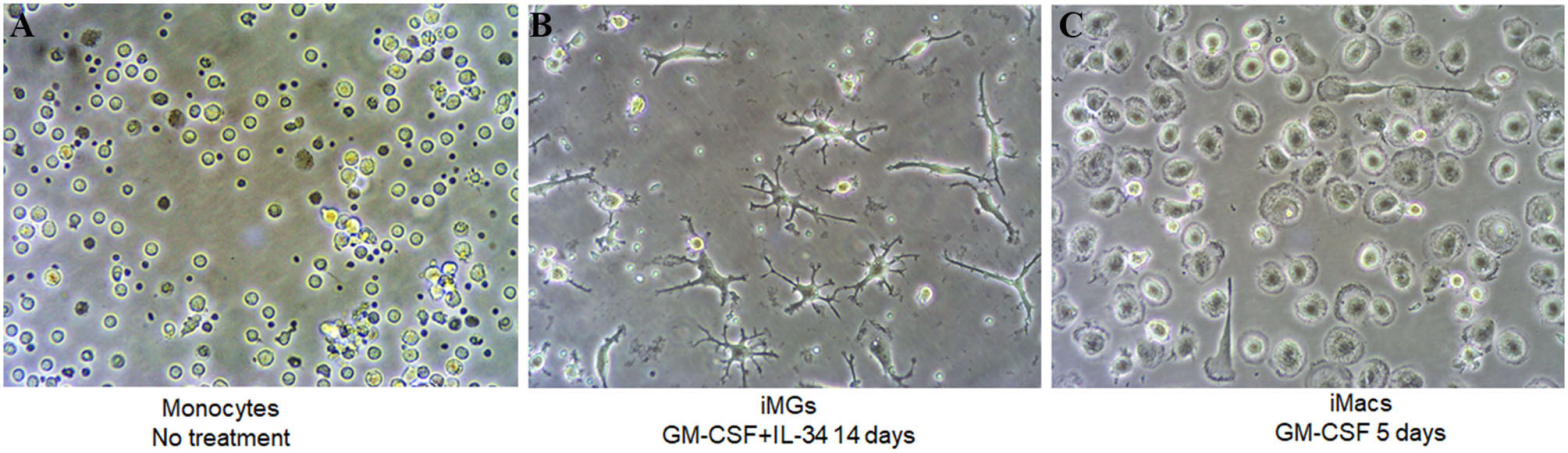
iMG cells and iMacs were induced from human peripheral blood monocytes. Human peripheral blood monocytes were isolated from blood and stimulated with IL-34 and GM-CSF for microglia-like cells and GM-CSF only for macrophages. Representative images **(A)** monocytes after adherence without any treatment, **(B)** iMG cells on day 14 after monocytes treated with GM-CSF and IL-34, and **(C)** iMacs on day 5 after monocytes treated with GM-CSF.

In this study, we used fresh or frozen PBMCs to generate human induced microglia-like cells (iMG cells) using a cocktail of IL-34 (100 ng/ml) and GM-CSF (10 ng/ml) as described previously (Ohgidani et al., 2014; Sellgren et al., 2017). Untreated monocytes showed a round morphology (Figure 1A). Upon induction, the microglia-like cells started to grow branches on day 5 (Supplementary Figure 1A). On day 10–14, they displayed a typical morphology of resting ramified microglia with a small cell soma and multiple branching processes (Figure 1B). It was worth noting that the iMG cells could survive up to 36 days when culture media was replaced every 3 or 4 days (Supplementary Figure 1B). For macrophage induction, we treated monocytes with GM-CSF for 5–7 days. On day 5, the phase-contrast image showed the typical “fried-egg” shape of macrophages with enlarged circular cell body (Figure 1C), which was consistent with the previous description (Jin and Kruth, 2016).

### Microglia-Specific Markers Expressed by iMG Cells

**Figure 2:**
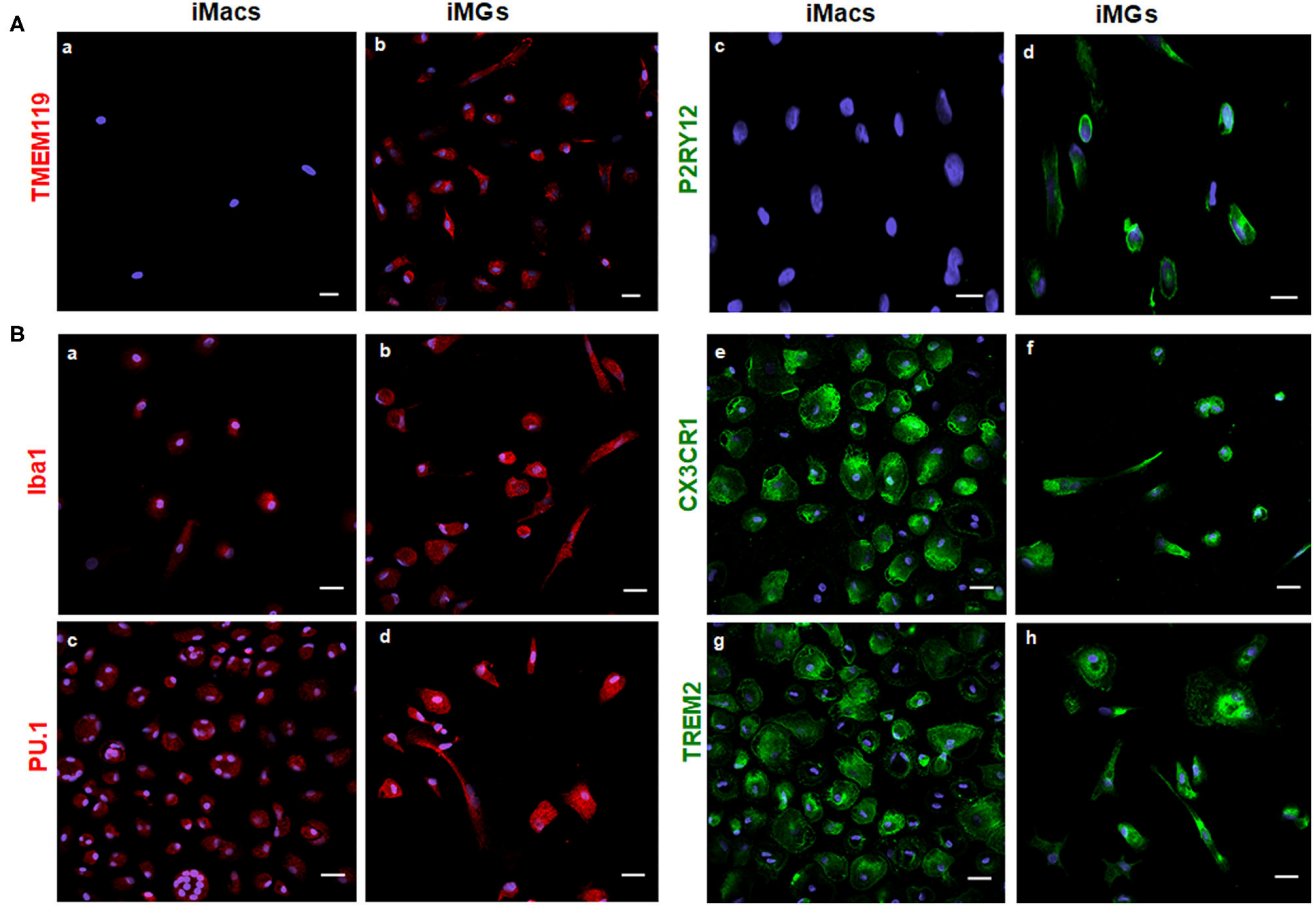
iMG cells expressed microglia-specific markers (TMEM119 and P2RY12) and other myeloid-specific markers. The expression of surface markers on the iMG cells and iMacs were performed by immunocytochemistry. Peripheral monocytes were incubated with GM-CSF (iMacs) for 5–7 days or cocktail of GM-CSF and IL-34 (iMG cells) for 10–14 days. Representative images of iMacs and iMG cells were shown. **(A)** iMG cells expressed unique microglial markers TMEM119 (b) and P2RY12 (d), which were absent from iMacs (a,c). **(B)** Myeloid lineage markers were expressed on both iMacs and iMG cells, such as Iba1 (a,b), PU.1 (c,d), CX3CR1 (e,f), and TREM2 (g,h). Images were captured at 40× with a scale representing 20 μm, where P2RY12, CX3CR1, and TREM2 were stained green, TMEM119, Iba1, and PU.1 were stained red. Cellular nuclei were stained blue with DAPI.

As a specialized macrophage, microglia share many common markers with macrophages, including Iba1 (Ionized calcium binding adapter molecule 1), transcription factor PU.1, chemokine receptor CX3CR1, and TREM2. On the other hand, transmembrane protein 119 (TMEM119) and purinergic receptor P2RY12 are considered unique markers for microglia but are absent on macrophages (Satoh et al., 2016; Zhu et al., 2017). In this study, we used both myeloid and microglia-specific markers to verify the specificity of iMG cells. As expected, TMEM119 and P2RY12 markers were expressed by iMG cells, as shown in Figure 2A(b,d). These two markers were absent on iMacs, as shown in Figure 2A(a,c). These results were consistent with recent studies from two different groups (Muffat et al., 2016; Douvaras et al., 2017), where hiPSC-derived microglia-like cells also expressed TMEM119 and P2RY12 markers. On the other hand, myeloid lineage markers, such as Iba1, PU.1, CX3CR1, and TREM2, were all expressed by both iMG cells s shown in Figure 2B(b,d,f,h) and iMacs as shown in Figure 2B(a,c,e,g). The results from immunocytochemistry indicated that monocytes-derived iMG cells and iMacs expressed common markers of myeloid lineage but only iMG cells expressed microglia-specific markers, TMEM119 and P2RY12. Therefore, TMEM119 and P2RY12 can be used as markers to distinguish iMG cells from monocyte-derived macrophages.

### Phagocytosis Assay in iMG Cells

**Figure 3:**
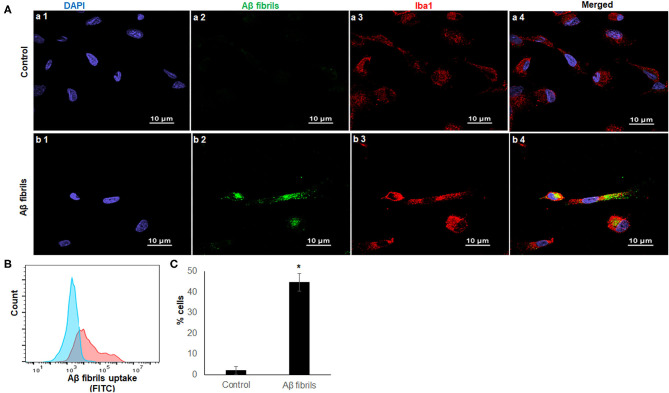
iMG cells phagocytosed fibrillary Aβ_42_. Confocal imaging and flow cytometry analysis of iMG cells were incubated with human HiLyte™ Fluor 488-labeled Aβ_42_ proteins. Phagocytosis activity was measured after 4 h incubation. **(A)** Representative confocal images were shown where controls cells were panel a (a1–a4) and iMG cells with phagocytosed fAβ_42_ were panel b (b1–b4). Images were captured at 100× with a scale representing 10 μm. fAβ_42_ and Iba1were stained green and red, respectively. Nuclei were stained blue with DAPI. Merged images of all three channels were shown in a4 and b4. Merged image (b4) indicated Aβ_42_ proteins were present within the cytoplasm of iMG cells. **(B)** The Aβ_42_ protein uptake was quantified by flow cytometry. Representative image of three experiments were shown, where the blue graph was for control and red for iMG cells with engulfed fAβ_42_. **(C)** Percentage of cells with engulfed fAβ_42_ as measured by flow cytometry. The result was from three independent experiments, and the error bars represented ± SD. *p ≤ 0.05 was considered as significant.

Phagocytosis is one of the most fundamental functions of microglia, and it maintains CNS homeostasis (Fu et al., 2014). Aβ_42_ are found abundantly in the AD brain, and fibrillary Aβ_42_ (fAβ_42_) is closely linked to AD pathogenesis (Crouch et al., 2008). To determine the phagocytic capacity of iMG cells, we used fAβ_42_ to mimic the physiological condition of AD patients_._ Considering that Iba1 is a microglia/macrophage-specific calcium-binding protein that participates in membrane ruffling and phagocytosis in activated-microglia, we co-stained the iMG cells with anti-Iba1 antibody to ensure that fAβ_42_ were physically engulfed inside the cells. As a negative control, no green fluorescent fAβ_42_ were detected inside iMG cells in Figure 3A(a1–a4), where the iMG cells were not treated with fAβ_42_. However, green fluorescent fAβ_42_ were detected inside iMG cells, along with the red fluorescent Iba1 staining in the cytoplasm, as seen in Figure 3A(b1–b4). The confocal images clearly indicated that iMG cells could phagocytose extracellular protein aggregates from their surroundings. Next, we quantitated the percentage of iMG cells with phagocytic activity by flow cytometry. Our result showed that 44.73 ± 4.20% of iMG cells had fluorescent-labeled fAβ_42_ (Figures 3B,C). Together, we showed that iMG cells indeed could engulf exogenous proteins (fAβ_42_), implicating potential application for AD study. With the flow cytometry for fluorescent fAβ_42_ detection, our model has the potential to measure and compare the microglial capacity between different groups, such as between AD patients and controls.

### M1 and M2 Phenotypic Changes in iMG Cells During Polarization

**Figure 4:**
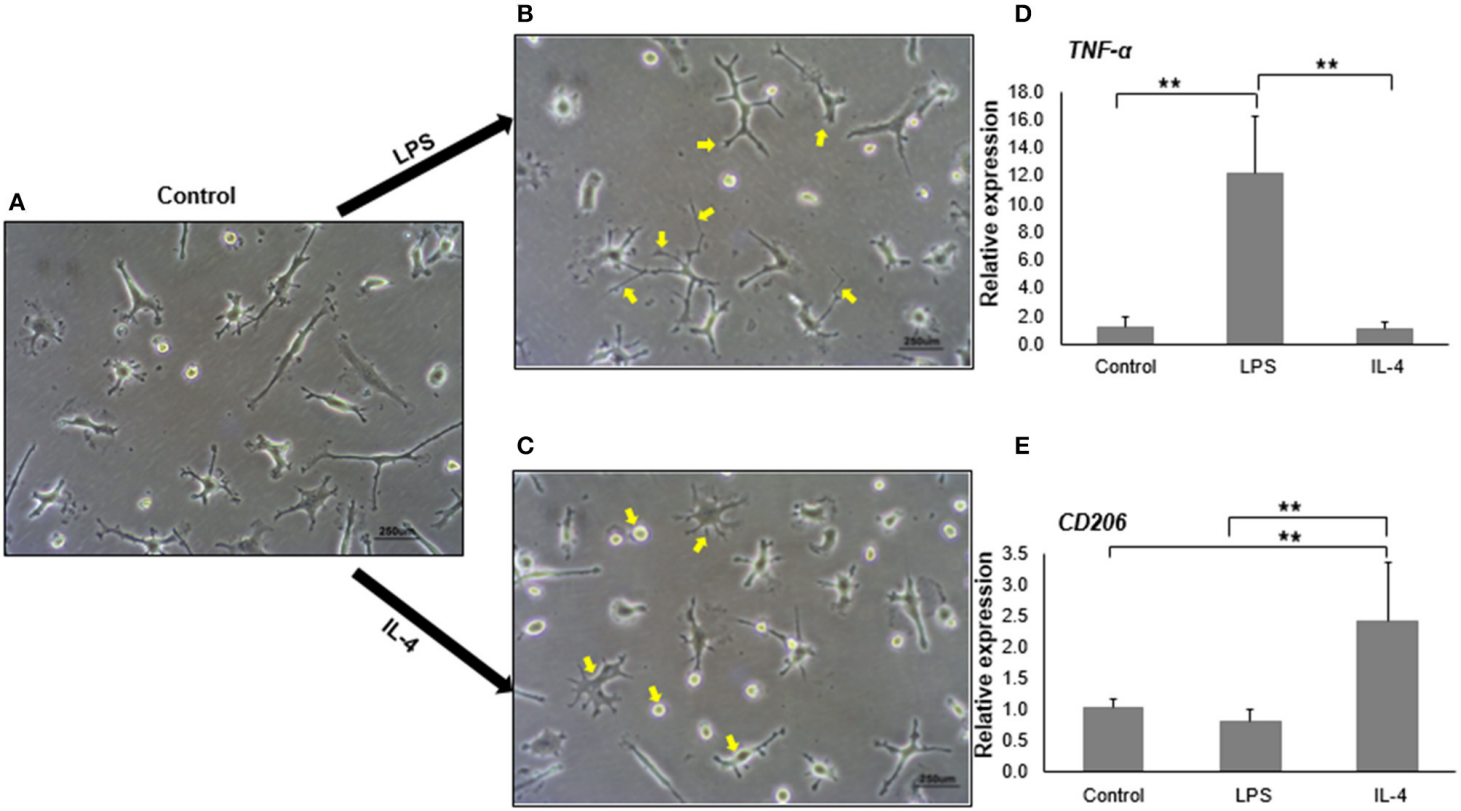
*In vitro* activation and polarization of iMG cells. The morphology of iMG cells was captured after stimulation with LPS or IL-4 for 24 h. **(A)** Control iMG cells without any treatment, **(B)** iMG cells stimulated with 10 μg/ml LPS showing hyper-ramification as indicated with arrows, **(C)** iMG cells stimulated with 20 ng/ml IL-4 showing ameboid structure as indicated with arrows. Changes in gene expression were analyzed by qRT-PCR showed **(D)** increased *TNF-*α levels in LPS-stimulated iMG cells and **(E)** increased *CD206* levels in IL-4-stimulated iMG cells. The experiments were all repeated at least three times. Data were represented as a mean ± SD. Significance between groups were determined by ANOVA test, where ** denoted *p* < 0.005.

Upon stimulation, microglia have the capacity to polarize toward a spectrum of activation states ranging from M1 (pro-inflammatory state) to M2 (anti-inflammatory state) (Tang and Le, 2016). Phenotypic changes, including morphology, gene expression, and cytokine release, occur during the polarization (Peng et al., 2017). LPS is a well-known agent for M1 polarization, whereas IL-4 is often used for M2 polarization. With LPS treatment, we noticed that M1 activated-iMG cells had undergone morphological changes showing super-ramified long branches (Figure 4B) compared to iMG cells without activation (Figure 4A). On the other hand, iMG cells activated by IL-4 treatment, exhibited an amoeboid shape, having a round cytoplasm with fewer short branches (Figure 4C). These changes were consistent with previous reports that microglia in M1-state encompassed long processes with ramified shapes, and microglia in M2-state possessed short and thick processes (Lecours et al., 2018; Geirsdottir et al., 2019).

We then tested whether M1 and M2 activated-microglia expressed specific activation markers during the polarization. *TNF-*α, *CD86, CD16/32*, and inducible nitric oxide (*iNOS*) are commonly used as markers for M1 activated-microglia (David and Kroner, 2011; Kobayashi et al., 2013). M2 activated-microglia, however, often shows upregulation of *CD163* or *CD206* (Cherry et al., 2014). In this study, we used the mRNA expression levels of *TNF-*α and *CD206* as signals for M1 and M2 activation, respectively. As expected, *TNF-*α mRNA expression in iMG cells was significantly upregulated (12.20 ± 4.00 folds, *p* < 0.005) when treated with LPS (Figure 4D). IL-4 treatment did not change *TNF-*α mRNA levels in the iMG cells. Similarly, *CD206* mRNA expression was increased (2.41 ± 0.90 folds, *p* < 0.005) in iMG cells with IL-4 treatment (Figure 4E). LPS did not change *CD206* mRNA expression in iMG cells. The upregulation for M1 or M2 specific genes and the typical morphological changes demonstrated that iMG cells were highly plastic that they were able to polarize into M1 or M2 state when responding to exogenous stimulants (LPS or IL-4).

### iMG Cells Closely Clustered With Brain-Resident Microglia From RNA-seq Analyses

**Figure 5:**
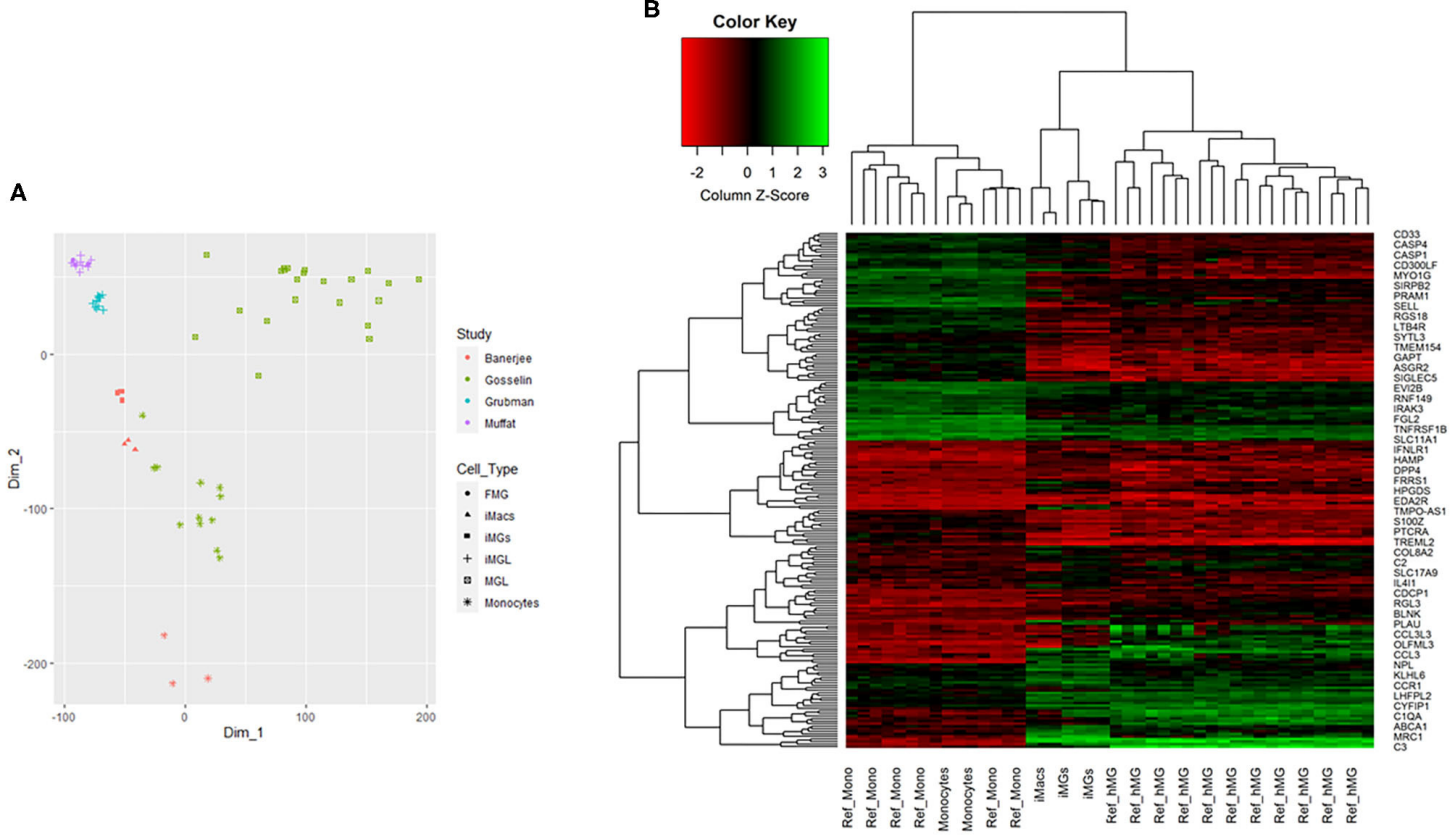
Multidimensional scaling analysis (MDS) and 2D hierarchical clustering from RNA-seq profile of iMG cells. **(A)** MDS analysis for multiple RNA-seq datasets, including iMG cells, iMacs, and monocytes from our study, hiPSC-induced microglia (iMGL) and fetal microglia (FML) from Muffat et al.'s study, iMGL from Grubman et al.'s study, and human brain microglia (MGL) and monocytes from Gosselin et al.'s study. **(B)** 2D hierarchical clustering with 201 top differentially expressed genes from our study and Gosselin et al.'s study, where Ref_hMG and Ref_Mono were human brain-resident microglia and monocytes from Gosselin et al.'s study (Gosselin et al., 2017). iMG cells were induced microglia-like cells from monocytes and iMacs were induced macrophages from monocytes in our study.

We showed that iMG cells were similar with primary microglia in morphology, specific markers, and functions. Now we asked whether the iMG cells were similar to human brain-resident microglia or hiPSC-induced microglia from their gene expression profile. For this purpose, we did RNA-seq for monocytes, iMacs, and iMG cells. We also downloaded RNA-seq data from Muffat et al. (2016), Gosselin et al. (2017), and Grubman et al. (2020) for comparison (see Supplementary Table 3 for more details about the datasets). We first conducted PCA analyses for the whole-genome RNA-seq data for the three groups of cells (monocytes, iMacs, and iMG cells) in our induction system. Indeed, we identified three distinctive groups for monocytes, iMacs, and iMG cells from the PCA plot (Supplementary Figure 2), suggesting that the monocytes were successfully differentiated into two other cell types (iMacs or iMG cells) with different induction cytokines. To further determine whether iMG cells resembled the brain-resident microglia or hiPSC-induced microglia, we obtained gene expression data from the above mentioned studies (Muffat et al., 2016; Gosselin et al., 2017; Grubman et al., 2020). We then conducted MDS analyses with normalized data. As shown in PCA plot (Supplementary Figure 2), the MDS plot clearly showed that the three groups of cells were well-separated in our studies (Figure 5A). hiPSC-induced microglia from Muffat et al. and Grubman et al.'s groups, while clustered by each study, there were some distances between the studies. The resident microglia and monocytes from the Gosselin et al.'s study were scattered. This might be due to the different micro-environment where these cells resided. Overall, iMG cells from our study were the closest to the hiPSC-induced microglia and the resident microglia from human brain, but they were separated from monocytes. This confirmed that our iMG cells did have the features of microglia when mRNA expression data were compared.

We further performed 2D hierarchical clustering analysis of the RNA-seq data from our iMG cells against previously published RNA-seq data from human brain-resident microglia (Ref_hMG) (Gosselin et al., 2017). As mentioned above, a total of 914 microglial signature genes were included in this study (Supplementary Table 2). After matching with our RNA-seq profiles, we obtained an overlap of 805 genes shared between iMG cells and Ref_hMG, among which 312 were consistent between the two groups, as compared with fold changes with monocytes. Based on further differential analysis across iMG cells and monocytes with criteria |FC| (fold changes) ≥1.2, *p* ≤ 0.001, and average expression ≥5, we generated top 201 microglial differentially expressed genes. With RNA profiles from iMG cells and Ref_hMG, we attempted to evaluate how close the induced-microglia resembled human brain microglia at RNA expression levels. With the top 201 microglial differential genes, we conducted 2D hierarchical clustering analysis. Our data showed that iMG cells clustered closely with Ref_hMG, whereas monocytes from our experiments clustered closely with Ref_Monocytes (Figure 5B). We also noticed that iMG cells and iMacs were clustered closely to each other. These results demonstrated that monocytes treated with IL-34 and GM-CSF underwent significant changes in gene regulation and differentiated toward human microglia-like cells. The expression pattern of surface markers were consistent with a previous report (Ohgidani et al., 2014; Sellgren et al., 2017). As compared to monocytes, the iMG cells and iMacs were clustered more closely, reflecting that iMG cells were specialized macrophages in the brain.

### Venn Analysis and Differential Analysis Across iMG Cells, iMacs, and Monocytes

**Figure 6:**
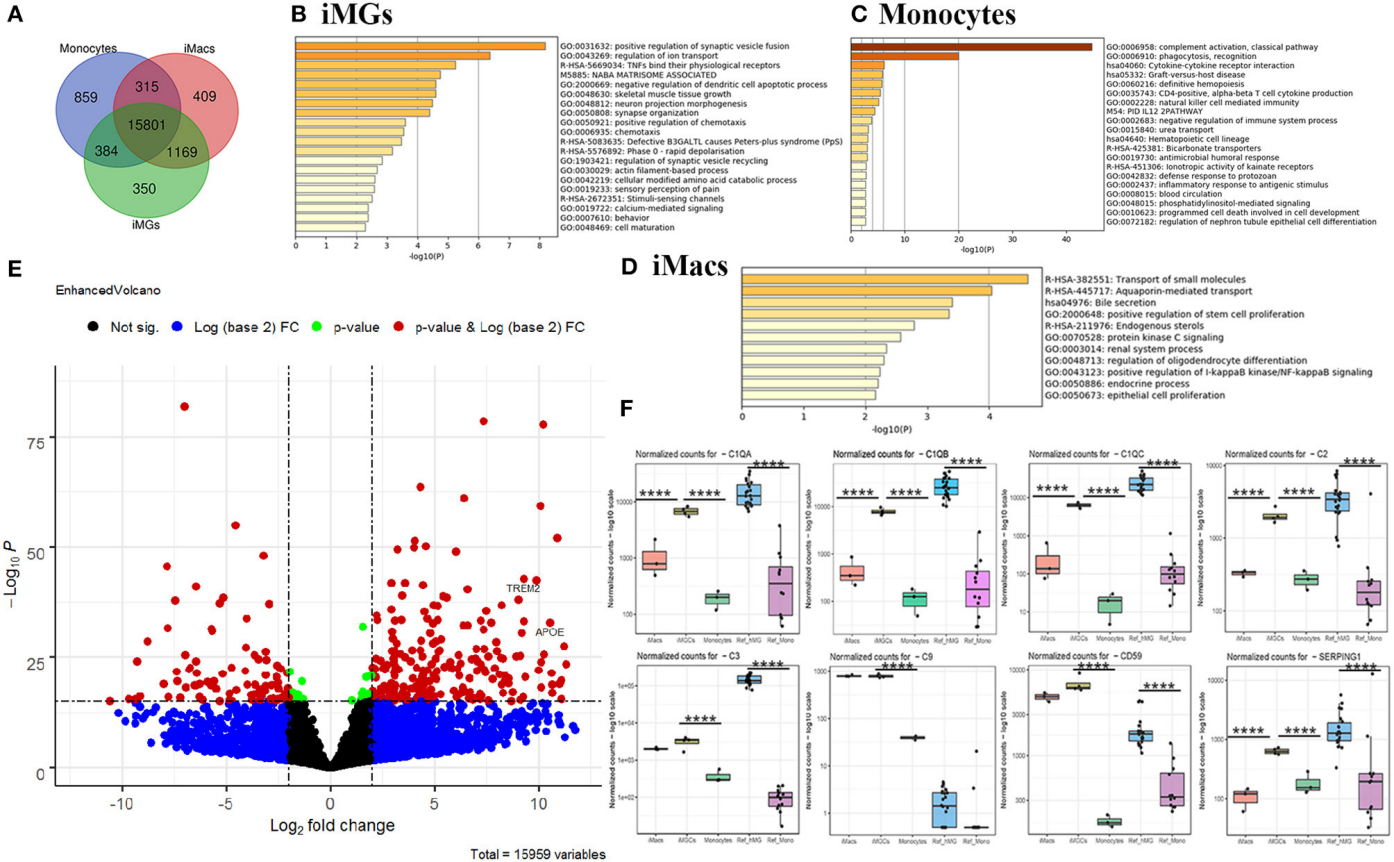
Venn and differential expression analysis and enrichment analysis. **(A)** Venn diagram of genes expressed by iMG cells, iMacs, and monocytes (defined by the mean of the normalized counts >10). Blue: Monocytes; Red: iMacs; Green: iMG cells. Enrichment analysis with the unique genes expressed in iMG cells **(B)**, monocytes **(C)**, and iMacs **(D)**, ranked by pathway *p*-value. **(E)** Volcano plot depicting differentially expressed genes in iMG cells against monocytes. The dashed line indicated *p* < 1 × 10^−16^ (horizontal line) and |log2FC| >2 (Vertical line). Two well-known AD risk genes, *APOE* and *TREM2*, were both upregulated in iMG cells as labeled in the Volcano plot. **(F)** Relative expression of significant genes (*C1QA, C1QB, C1QC, C2, C3, C9, CD59*, and *SEPRING1*) from the complement system in iMG cells vs. monocytes, and iMacs and. *p* values were obtained from differential expression analysis DESeq2 v1.28.1, where **** denotes *p* < 0.001.

To comprehend the differences between monocytes, iMacs, and iMG cells, we performed Venn analysis across the three groups. Unique genes to each cell type and shared genes across three or two cell types were shown in the Venn diagram (Figure 6A), where 350 genes were uniquely expressed in iMG cells. We also found that 409 genes and 859 genes were specifically expressed in iMacs and monocytes, respectively. 15,801 genes were shared across all three groups.

Gene enrichment analysis was performed with unique genes in each group. Our results showed that the unique genes from iMG cells were enriched in the pathways involving in the regulation of synaptic vesicle fusion and ion transport, and TNFs bind their physiological receptors, which aligned with microglial function in regard to their interaction with neurons and response to the environment (Figure 6B). The unique genes from monocytes were enriched in innate immune-related pathways, such as complement activation, phagocytosis, and cytokine-cytokine receptor interaction (Figure 6C). Surprisingly, 409 unique genes from iMacs were not enriched in many pathways, but only associated with the transport of small molecules pathway (Figure 6D).

The differences in gene expression between iMG cells and monocytes were also shown in the volcano plot, where significant genes were those that passed the FDR (*p* < 1 × 10^−16^) and |log2FC| > 2, as indicated in red (Figure 6E). Interestingly, *APOE* and *TREM2*, two of the most important risk genes for AD, were both significantly upregulated (Figure 6E), with *p* = 7.09 × 10^−31^ and 2.35 × 10^−40^, respectively. Differentially expressed genes (DEGs) between iMG cells and monocytes, or Ref_hMG and monocytes, were shown in Supplementary Tables 4, 5.

Genes in the complement system are highly expressed in microglia and play an important role in synaptic pruning that may underlie the mechanisms of brain diseases, such as schizophrenia and AD. Ryan et al.'s group recently reported a microglia-like model generated from human monocytes in the presence of polarizing cytokines (IL-34, GM-CSF, M-CSF, NGF-β, and CCL2). We wanted to compare some of the microglia-specific genes from those of previous studies. Our data showed that genes involved in the synaptic remodeling, such as complement factors *C1QA, C1QB, C1QC, C2, C3, C9, C59*, and *SERPING1*, were highly expressed in our iMG cells, consistent with Gosselin et al.'s report and Ryan et al.'s report (Figure 6F) (Gosselin et al., 2017; Ryan et al., 2017). Among those, *C1QA, C1QB, C1QC*, and *C2* were only upregulated in iMG cells but not iMacs. We also found that more than half of genes (19) in iMG cells overlapped with 37 genes from MDMi (human monocyte-derived microglia-like cells) from Ryan et al.'s group (Ryan et al., 2017) (Supplementary Table 6). Interestingly, four genes mentioned as being microglia-specific in mice in Ryan et al.'s report, *TGF*β*R1, PROS1, P2RX7*, and *C1QB*, were consistently upregulated in our iMG cells (Supplementary Figure 3). As mentioned above, two of the most important risk genes for AD, *APOE* and *TREM2*, were also significantly upregulated (Supplementary Figure 3).

## Discussion

Microglia are essential for the maintenance of normal brain functions. Massive evidence shows that microglial dysfunction contributes to neurodevelopmental and neurodegenerative processes. Over the past 100 years of research on microglia, advances have been made in understanding microglial plasticity, transcriptional diversity, function-related signaling pathways, and stem-cell-based microglial induction systems (Umpierre and Wu, 2020). Technical developments also have advanced scientists to explore human microglia-like cellular models from human blood monocytes (Ohgidani et al., 2014; Ryan et al., 2017; Sellgren et al., 2019), or hiPSC (Muffat et al., 2016; Abud et al., 2017; Douvaras et al., 2017; Haenseler et al., 2017; Pandya et al., 2017; Svoboda et al., 2019). *In vitro* differentiation from hiPSC to microglia is challenging as the reprogramming process is time-consuming (Liang and Zhang, 2013). It is essential to establish a more efficient microglial model closely resembling the human microglial phenotypic and functional features. It is also crucial to build the model from a relatively easy and non-invasive sample collection process. With modification from Ohgidani et al.'s report (Ohgidani et al., 2014), we generated and validated the microglia-like model from human peripheral monocytes. Consistent with the previous studies (Ohgidani et al., 2014; Sellgren et al., 2017), our data showed that iMG cells had the typical ramified morphology as resting microglia in the brain and expressed microglia-specific surface markers, such as TMEM119 and P2RY12. On the contrary, iMacs with GM-CSF treatment showed a typical “fried-egg” morphology without TMEM119 and P2RY12 expression. Another group has used a complex cocktail (IL-34, GM-CSF, M-CSF, NGF-β, and CCL2) for microglia induction (MDMi) (Ryan et al., 2017). While comparing microglial genes, iMG cells and MDMi shared more than half of those microglia-specific genes and 4 specific genes were significantly upregulated in iMG cells, MDMi, and human microglia. These results demonstrated that microglia induced from monocytes with different inductions shared some common microglia features, though, at this stage it is not clear what the difference is between the two inductions. We know that differentiation from hiPSC to microglia-like cells usually takes around 40 days (Muffat et al., 2016; Grubman et al., 2020). In this study, our induction from monocyte to microglia took <2 weeks. In addition, blood collection from patients is widely accepted for clinical research. Thus, there is no doubt that our model will provide a more feasible and efficient approach to study microglial functions in clinical trials.

As mentioned above, microglia are professional phagocytic cells of the brain that remove apoptotic or necrotic cells (Green et al., 2016) and eliminate unfolded proteins, such as Aβ (Daniel Lee and Landreth, 2010). To evaluate the phagocytic capacity of iMG cells, we chose fibrillary Aβ as an exogenous proteins to best mimic the physiological condition for phagocytosis in most AD patients. Nonetheless, it should be noted that different Aβ preparation methods can affect Aβ aggregation kinetics and structures (Yates and Legleiter, 2014; Nirmalraj et al., 2020). It is still not clear under what circumstances various types of Aβ are formed physiologically inside the brain (Chen et al., 2017). Microglia are believed to initially clear Aβ deposits at a preclinical stage of AD. With the progress of AD, they somehow lose their ability to clear Aβ as they are activated to a pro-inflammatory state where cytokines, chemokines, and reactive oxygen species (ROS) are released (Hale et al., 2008). To our knowledge, we are the first to show the phagocytic capacity of monocyte-derived microglia engulfing exogenous Aβ proteins. This is crucial as utilizing this microglial model will help us to understand the underlying mechanism by which microglia remove Aβ proteins under physiological condition and how they lose the ability of Aβ clearance during pathological condition. Our cellular model provides a feasible tool to measure microglial function of phagocytosis. With different compounds or with genetic engineering approaches, iMG cells can be used to evaluate their effects on removing internal or external proteins, including Aβ proteins in AD clinical trials. As a result, this model would offer a prompt monitor system for microglial functions at the cellular level, which facilitates new drug discovery or disease-modified treatment.

Microglia are highly plastic; they can be activated into the pro-inflammatory (M1) state or into the anti-inflammatory (M2) state in response to various stimuli. Such microglia remodeling is critical for microglial phagocytosis, synaptic modifications, and neuroinflammatory responses and normal brain functioning. As an example, pro-inflammatory M1 microglia are usually dominant in AD and schizophrenia pathology. With morphological changes and specific polarization markers upregulation, our data demonstrated that iMG cells could be transformed into M1 state with LPS stimulation or M2 state with IL-4 stimulation. Thus, iMG cells provide a human microglial cellular model that can be used to study the mechanisms underlying the microglial polarization (Svoboda et al., 2019). Future studies with the iMG cells model for comprehensive comparison between patients and healthy controls have the potential to identify the microglia-relevant mechanisms associated with AD, schizophrenia, and other diseases.

In this study, we also conducted a comprehensive gene expression profiling of iMG cells and compared it with expression data from brain-derived microglia. From cluster analysis, our data indicated that iMG cells were closely clustered with brain-resident microglia or hiPSC-induced microglia. Interestingly, the unique genes from iMG cells were enriched in pathways involved in the regulation of synaptic vesicle and ion transport, and synapse organization. Multiple genes in the complement system, *C1QA, C1QB, C1QC, C2, C3, C9, CD59*, and *SEPRING1*, were also highly up-regulated. This confirms that microglia-specific genes had been remodeled during the differentiation from monocytes to iMG cells and possess specialized brain functions (e.g., synaptic remodeling). Excessive evidence has shown that microglial dysfunction contributes to synaptic abnormalities and cognitive decline, which are often seen in neurodevelopmental and neurodegenerative disorders. Indeed, Sellgren et al. recently applied the model to investigate schizophrenia. They found that iMG cells derived from patients with schizophrenia eliminated more synapses than those from healthy controls (Sellgren et al., 2019). Sellgren et al. demonstrated that iMG cells would be a good model to study schizophrenia. Several other groups have successfully applied this iMG model or similar model to study the microglial functions in different disorders, including bipolar disorder, fibromyalgia, and schizophrenia (Ohgidani et al., 2017a,b; Ormel et al., 2020). In this study, we demonstrated that the iMG cellular model would also be a good model for studying cellular changes and gene regulation during the development of AD, mainly the phagocytosis of fAβ_42_ and the gene expression profiles. The two genes *TREM2* and *APOE* are widely accepted as high risk genes for non-familial AD (Shi and Holtzman, 2018; Wolfe et al., 2018). Our RNA expression data indicated the two genes were significantly upregulated in iMG cells as compared to monocytes.

Our iMG cellular model has some limitations. In spite of the promising results, the model might still not fully recapitulate the microglial phenotypes from the human brain. We understand this limitation and acknowledge that this is a challenge to any surrogate cellular model in the field. Nevertheless, our study showed that the iMG cellular model had extensive human microglial features at phenotypic, functional, and gene expression levels that warrant its application in schizophrenia and AD studies. Other limitations include subject to subject variations, cell viability during induction, and high sensitivity to culture microenvironments. We acknowledge that these limitations may create some variations across batches of experiments. However, the results from our *in vitro* iMG cells were highly compatible and consistent with studies from other groups. Therefore, our model derived from patients with psychiatric diseases or neurodegenerative diseases would be a good alternative for the study of gene regulations and characterization of disease-related microglial phenotypes, provided that each experiment has age- and sex-matched controls. Furthermore, our study indicated that this microglial model can be extended to study the molecular mechanisms underlying synapse remodeling during brain development and neurodegeneration. Finally, this iMG model can also be used for high throughput studies for the discovery of new biomarkers or new drugs.

Overall, our findings confirmed that iMG cellular model exhibited cellular and molecular characteristics that were similar to human brain-resident microglia. Functional studies of such patient-specific microglial model will facilitate a better understanding of neuroinflammatory processes in psychiatric and neurodegenerative diseases. We were the first to validate the iMG cellular model with comprehensive RNA profile, although we were not the first study to generate this human monocyte-derived microglial model. Additionally, this study investigated the plasticity of the induced-microglia with two distinct polarization states. Therefore, the present study provides valuable data regarding the application of this model to study the pathogenesis of psychiatric and neurological disorders, including schizophrenia and AD, two diseases with cognitive deficits closely linking to microglial activation, synaptic pruning, and neuroinflammation. Further studies using this cellular model derived from patient samples are required to confirm our findings.

## Data Availability Statement

The whole genome RNA-seq project PRJNA678841 generated during the current study in the format of fastq files have been deposited at the National Center for Biotechnology Information (NCBI) repository (http://www.ncbi.nlm.nih.gov/bioproject/678841).

## Author Contributions

JC conceived the idea. JC and AB conducted the experiments and analyzed the data. KD performed the qRT-PCR. TM collected and processed the RNA-seq raw data in this study. JC, YL, and XW conducted the RNA-seq and bioinformatics analysis. JC, AB, and XC contributed to the writing of the manuscript. All authors reviewed the manuscript and agreed on its content.

## Conflict of Interest

XC is employed by 410 AI, LLC. The remaining authors declare that the research was conducted in the absence of any commercial or financial relationships that could be construed as a potential conflict of interest.

## Supplementary Material

Supplementary Figure 1 The morphology of iMG cells on days 5 and 36. **(A)** Branched cells were observed on day 5 after the treatment with IL-34 and GM-CSF. **(B)** The iMG cells survived up to 36 days.

Supplementary Figure 2 Principal component analysis (PCA) from RNA-seq profile. PCA plot of monocytes, iMacs, and iMG cells from whole-genome RNA-seq profile.

Supplementary Figure 3 Upregulation of microglia-specific genes and AD risk genes in iMG Cells. Four genes mentioned as being mice microglia-specific in Ryan et al.'s report, *TGF*β*R1, PROS1, P2RX7*, and *C1QB*, were consistently upregulated in our iMG cells. Two AD-risk genes, *APOE* and *TREM2*, were significantly upregulated in iMG cells. *P*-values were obtained from differential expression analysis DESeq2 v1.28.1., where ^****^ denotes *p* < 0.001, ^***^ denotes *p* < 0.005.

Supplementary Table 1 Sample information for RNA-seq profiling.

Supplementary Table 2 Microglia signature genes in this study.

Supplementary Table 3 Datasets for MDS analysis in this study.

Supplementary Table 4 DEGs between iMG cells and monocytes.

Supplementary Table 5 DEGs between Ref_hMG and monocytes.

Supplementary Table 6 Overlapping genes between iMG cells and Ryan et al.'s data or Ref_hMG and Ryan et al.'s data.
