# Supplementary figures and images for: Validation of Induced Microglia-Like Cells (iMG Cells) for Future Studies of Brain Diseases
Source: Front Cell Neurosci. 2021 Apr 9;15:629279. doi: 10.3389/fncel.2021.629279 (PMC8063054; doi:10.3389/fncel.2021.629279)

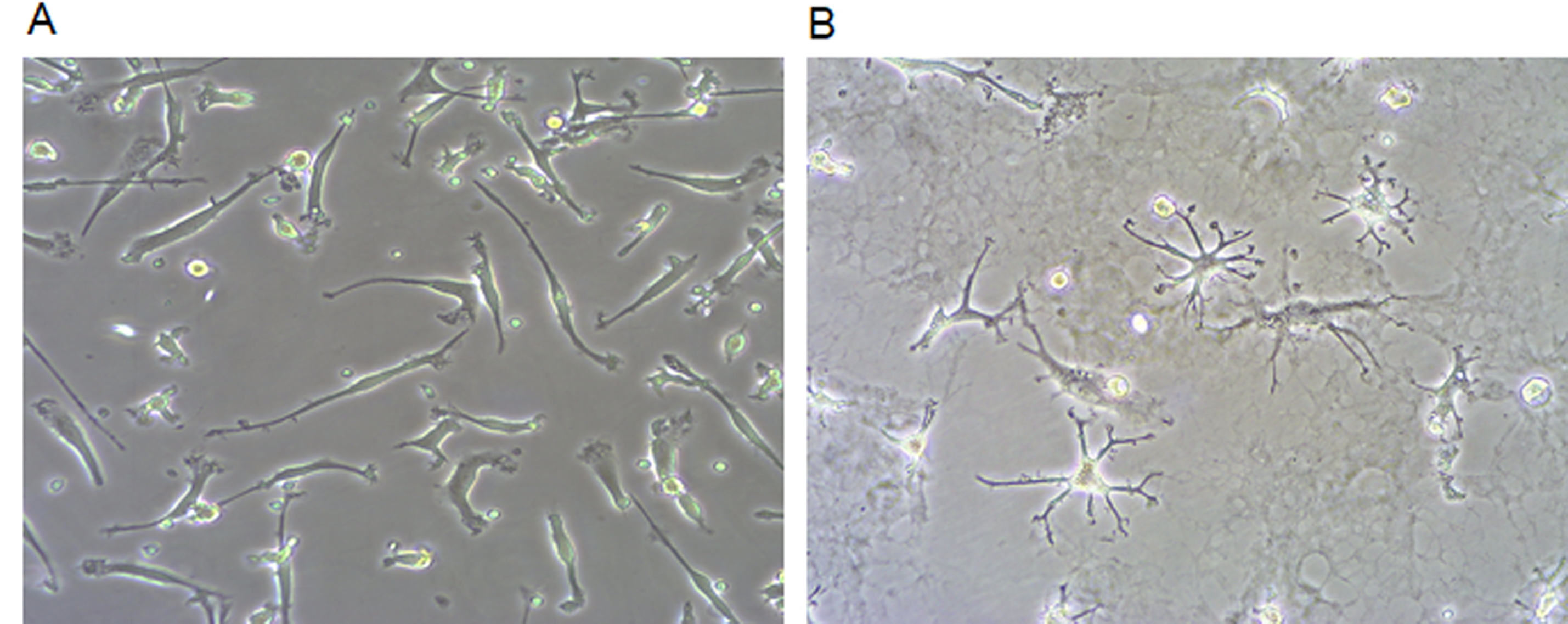

Supplement: Supplementary Figure 1 — The morphology of iMG cells on days 5 and 36. (A) Branched cells were observed on day 5 after the treatment with IL-34 and GM-CSF. (B) The iMG cells survived up to 36 days. [file Image_1.TIF]

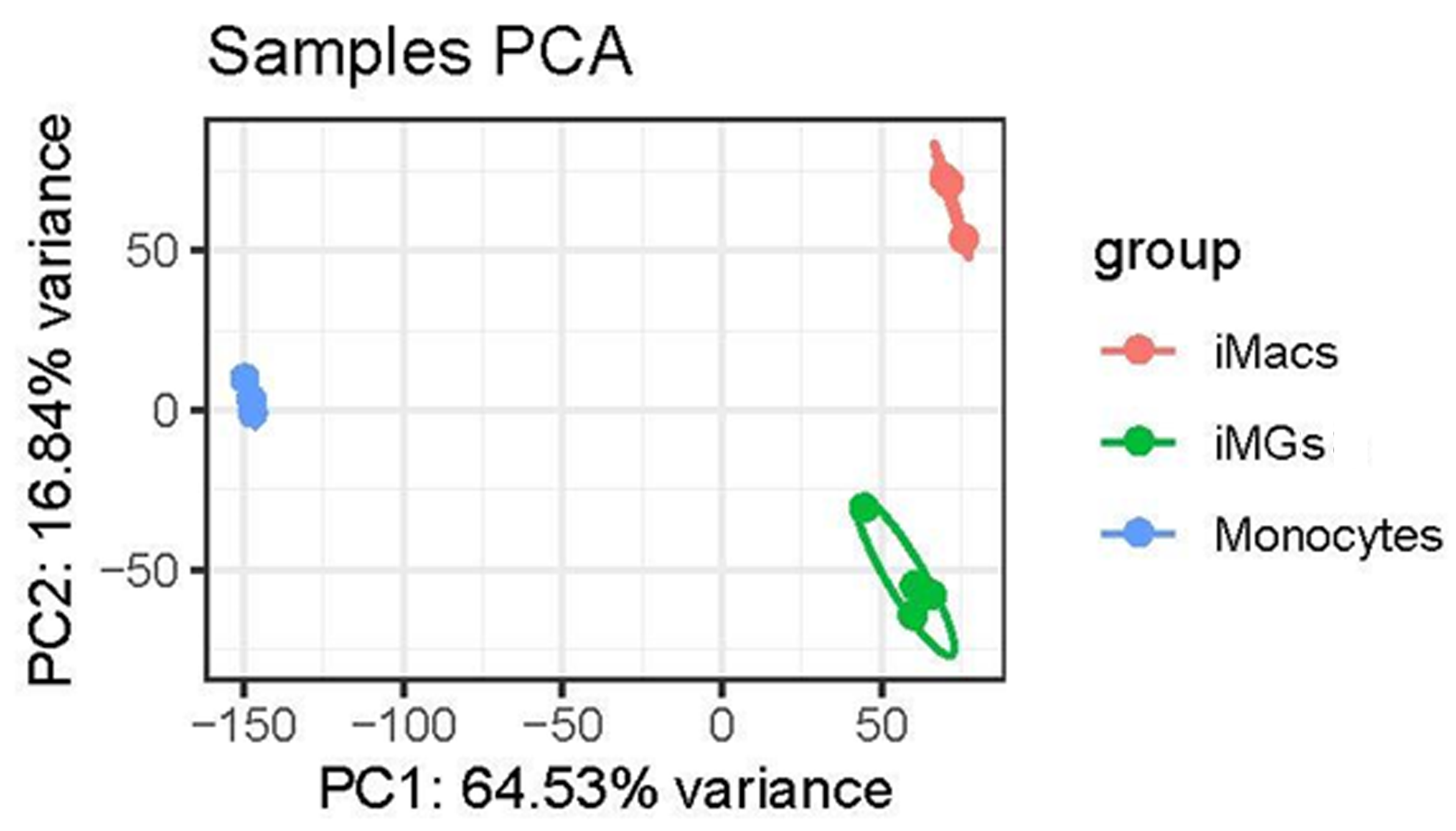

Supplement: Supplementary Figure 2 — Principal component analysis (PCA) from RNA-seq profile. PCA plot of monocytes, iMacs, and iMG cells from whole-genome RNA-seq profile. [file Image_2.TIF]

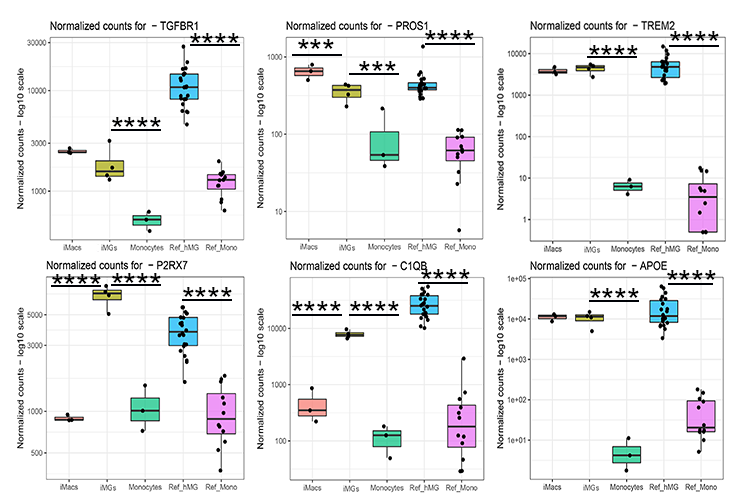

Supplement: Supplementary Figure 3 — Upregulation of microglia-specific genes and AD risk genes in iMG Cells. Four genes mentioned as being mice microglia-specific in Ryan et al.'s report, TGFβR1, PROS1, P2RX7, and C1QB, were consistently upregulated in our iMG cells. Two AD-risk genes, APOE and TREM2, were significantly upregulated in iMG cells. P-values were obtained from differential expression analysis DESeq2 v1.28.1., where **** denotes p < 0.001, *** denotes p < 0.005. [file Image_3.TIF]
